# The effect of *Methylococcus capsulatus* in mono- or co-culture with *Methanobrevibacter smithii* or with mixed rumen fluid on bacterial growth and methane gas production

**DOI:** 10.1093/jas/skaf383

**Published:** 2025-11-03

**Authors:** Byeng R Min, Hossam Ismael, Santosh Chaudhary, Mariline Hilaire, Vivian Kanyi, HongHe Wang, Heba Abdo, Ryszard Puchala

**Affiliations:** Department of Agricultural and Environmental Sciences, Tuskegee University, Tuskegee, AL 36088; Department of Agricultural and Environmental Sciences, Tuskegee University, Tuskegee, AL 36088; Department of Agricultural and Environmental Sciences, Tuskegee University, Tuskegee, AL 36088; Department of Agricultural and Environmental Sciences, Tuskegee University, Tuskegee, AL 36088; Department of Agricultural and Environmental Sciences, Tuskegee University, Tuskegee, AL 36088; Department of Agricultural and Environmental Sciences, Tuskegee University, Tuskegee, AL 36088; Department of Agricultural and Environmental Sciences, Tuskegee University, Tuskegee, AL 36088; American Institute for Goat Research, Langston University, Langston, OK 73050

**Keywords:** fermentation, *Methylococcus capsulatus*, microbiota, rumen

## Abstract

Providing an alternate electron sink to methane (CH_4_) is a crucial step in reducing enteric CH_4_ emissions. Comparing the effects of CH_4_-utilizing methanotroph bacteria (*Methylococcus capsulatus*; MC) and its combination with pure strains of rumen bacteria or mixed rumen fluid can offer biological insights into methanogenesis pathways and CH_4_ consumption. The primary objectives of this study were to investigate the impact of inoculating with *M. capsulatus* on the growth rates of rumen bacteria, including methanogens and mixed rumen fluid, as well as fermentation rates, ruminal gas production, CH_4_ emissions, and other environmental-impacting gases (N_2_O, H_2_S). Three experiments were carried out using in vitro ANKUM gas production systems (Exp. 1 and 2) and continuous recirculating flux chamber systems (Exp. 3). In Exp. 1, four strains of rumen bacteria—*Streptococcus bovis* [SB], *Ruminococcus flavefaciens* [RF], *Methanobrevibacter smithii* [MS], and *M. capsulatus* [MC]—were used to determine the effect of CH_4_-utilizing bacteria (e.g., MC) on specific growth rate, volatile fatty acid (VFA) production, and ruminal CH_4_ emissions in a combination with these bacterial strains. Results from Experiment 1 showed that MS produced the most CH_4_ among the strains. When cocultured with MC, no CH_4_ was detected, indicating that MC could utilize most of the CH_4_ produced in coculture with MS and other bacterial strains. There was little difference in total and cumulative gas production with varying MC doses (Exp. 2). However, in the presence of MC, CH_4_ production (percentage or g DM) decreased significantly (*P *< 0.01) as MC addition increased. Conversely, substrates containing both grain- and forage- based diets with MC increased N_2_O emissions per gram of DM (µg/g DM) or total N_2_O production (ppm), with treatment and basal diet interactions (*P *< 0. 01). In Exp. 3, using a continuous recirculating flux chamber system, CH_4_ flux significantly reduced (*P *< 0.001) over time in both basal diets with MC inoculum. However, fermentation rates varied between treatments and diets. These findings demonstrate that adding MC inoculum to in vitro rumen fermentation chambers significantly reduces CH_4_ emissions compared to controls.

## Teaser

Our findings showed that *Methanobrevibacter smithii* increased nitrous oxide (N_2_O) emissions per gram of DM (µg/g DM) or total N_2_O during 24 h (ppm), with a significant interaction between treatment and basal diet (*P *< 01; Table 4). The rumen fermentation rate, as measured by total volatile fatty acids (VFA), was similar across treatment groups. Using a continuous recirculating flux chamber system, CH_4_ production over time in both forage- and grain-based diets with the *M. capsulatus* inoculum to in vitro rumen fermentation chambers significantly reduces CH_4_ was detected, suggesting that *M. capsulatus* emissions in ruminant animals or the agricultural compost industry. Such a mitigation strategy could influence nutrient metabolism and CH_4_ dynamics, underscoring the importance of dietary approaches in optimizing rumen function and productivity in ruminant animals.

## Introduction

*Methylococcus capsulatus* is a methanotroph that plays an important role in producing animal feed. Methanotrophs are prokaryotic organisms that use methane (CH_4_) as their main source of carbon and energy (Oremland and Culbertson, 1992). They are bacteria or archaea that can grow with or without oxygen (O_2_) and need single-carbon compounds to survive (Oremland and Culbertson, 1992). It is used commercially to produce animal feed from natural gas, particularly for monogastric species such as pigs, chickens, mink, foxes, and various fish species. The bacterium’s ability to grow well in low-oxygen environments and its thermotolerance make it suitable for these applications. While *M. capsulatus* is used to produce animal feed, there is limited research on its potential use as a CH_4_ mitigation option or food ingredient. In addition, only minimal studies have been conducted on the use of methanotrophic fermentation activity, combined with *M. capsulatus*, as a CH_4_ mitigation option in ruminants.

Thermodynamically favorable conditions for the growth of gut microorganisms have been reported. Many gut fermentative bacteria produce fermentation end products, including volatile fatty acids (VFA) and gases such as hydrogen (H_2_), CH_4_, and other trace gases (Suarez et al., 1998). These products allow metabolic flexibility in response to changes in the redox balance, providing mutual benefits to the microbiota. Different metabolic pathways lead to variations in carbon and electron flow, energy yield from substrates, and the final fermentation products (Nakamura et al., 2010). Cellulose-degrading bacteria and protozoal species generally release H_2_ electrons (Latham and Wolin, 1977; Min et al., 2006), which are oxidized by methanogens (e.g., *Methanobrevibacter smithii*), producing CH_4_ using H_2_ in the rumen (Moss et al., 2000). Four moles of H_2_ are needed to produce one mole of CH_4_ through the methanogenesis pathways for H_2_ disposal (Nakamura et al., 2010). Additionally, methanotrophs, also called methanophiles (e.g., *Methylococcus capsulatus*; MC), can utilize CH_4_ as their primary carbon and energy source (Bodrossy and Kovacs, 1994). This process occurs under limited aerobic conditions, involving monooxygenase enzymes in a feedback mechanism. Methanogens and methanotrophs are essential bacteria that regulate CH_4_ balance in nature and work synergistically—methanogens produce CH_4_, which methanotrophs use as their carbon and energy source. This concept is also applied in industrial practices such as livestock management, solid waste processing, and wastewater treatment. However, our understanding of the physical and chemical interactions between these thermodynamic processes, methanotroph activity, the rumen microbiome, and CH_4_ sinks *in vitro* remains limited. There is little information in the literature regarding the extent of CH_4_ catabolism by *M. capsulatus* associated with rumen microbes and how extensively this pathway is utilized.

## Materials and Methods

The experiment was conducted at the Caprine Research and Education Unit at Tuskegee University, Tuskegee, AL. The Tuskegee University Animal Care and Use Committee approved the animal care, handling, and sampling procedures for this experiment (#T02-2002-1).

### Experimental design

The primary objectives of this study were to investigate the impact of *M. capsulatus* inoculation on the growth of other bacteria, including methanogens and mixed rumen fluid, and to assess the fermentation rate, ruminal gas production, CH_4_ emissions, and the production of other gases, such as N_2_O and H_2_S. Three experiments were performed: in vitro ANKOM gas production systems (Exp. 1 and 2) and continuous recirculating flux chamber systems (Exp. 3). These experiments evaluated the inclusion of MC in pure culture (Exp. 1) or mixed rumen fluid (Exp. 2 and 3) combined with grain- and forage-based diets, focusing on bacterial growth rate, rumen fermentation, and ruminal CH_4_ production. The bottles were flushed with CO_2_ before being sealed with an ANKOM Gas Production module. One-liter FlexFoil PLUS sample bags (SKC Inc., Eighty-Four, PA) were attached to the modules. The bottles were incubated in a shaking incubator at 39°C for 48 h—bottles containing only inoculum and buffer served as blanks.

#### Animals and feeding management

Three individual rumen-cannulated steers (Black Angus, *Bos taurus*; body weight = 804.6 ± 37.3 kg) were selected as donors based on similar body weight. Rumen fluid was collected from each of the three steers 2 h after they were fed. Cattle had been receiving a free choice of Bermudagrass hay with feedlot ration (1.0% body weight) at around 8:00 am based on steam-flaked corn (52%), wet distiller’s grain soluble (WDGS; 20%), wet corn gluten (10%), cotton-seed meal (10%), chopped corn stalks (7%), and mineral and vitamin premix (1%). All the rumen-cannulated steers were maintained in 20 × 10 m cattle pens. Water and mineral salt blocks were freely accessible. Ruminal contents from individual steers were placed into pre-warmed, insulated thermal bottles, transported to the laboratory, homogenized, filtered through four layers of cheesecloth, and pooled in equal amounts of rumen fluid (1.0 L each) for use as an in vitro inoculum. Rumen fluid was maintained in a water bath at 39°C with CO_2_ saturation until inoculation (Min et al., 2019a).

#### Experimental procedures

In Experiment 1 (Hungate tube system), four strains of rumen bacteria—*Streptococcus bovis* (SB), *Ruminococcus flavefaciens* (RF), *Methanobrevibacter smithii* (MS), and *M. capsulatus* (MC)—were used to assess the effect of CH_4_-utilizing bacteria (MC) on specific growth rate, volatile fatty acids (VFA) production, and ruminal CH_4_ production in a combination with the other pure strains of bacteria.

**Figure 1.**
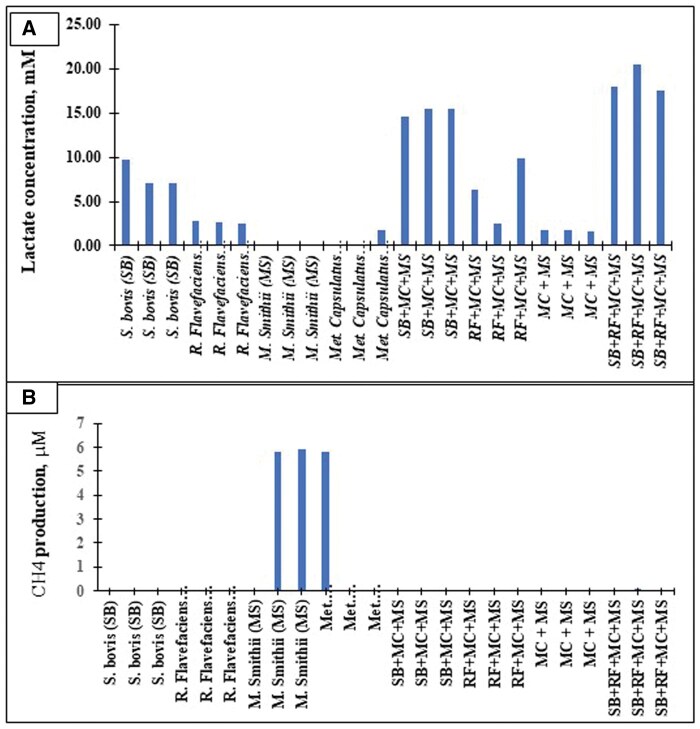
Rumen fermentation products: lactate (mM; a) and methane (CH_4_; b) gas production in the culture medium by predominant amylolytic (*Streptococcus bovis*; SB) and cellulolytic (*Ruminococcus flavefaciens*; RF) rumen bacterial pure culture of mono or mixed cultures with *Methylococcus capsulatus* (MC) and *Methanobrevibacter smithii* (MS) (*n* = 3).

For this objective, four pure strains were utilized individually or in various combinations with bacterial strains, as follows: SB, RF, MS, MC, MC + MS, SB + MC + MS, RF + MC + MS, and SB + RF + MC + MS. For validation of bacterial growth in monoculture or mixed cultures, at the end of the incubation period, the concentrations of lactic acid (mM; a) and methane gas emissions (µM/mL; b), were determined as a fermentation end product (Figure 1). In this study, CH_4_-producing archaea, *M. smithii*, were chosen as a suitable CH_4_ source for *M. capsulatus.* These four rumen bacteria were selected because they are essential bacteria contributing indirectly to rumen fermentation processes (Hua et al., 2022), as well as producing H_2_ from cellulose-degrading bacteria to support CH_4_ production, as described by Min et al. (2006). The pure strains of *M. capsulatus* were kindly provided by Dr Lori Giver, Feed/Kind/CALYSTA (1900 Alameda de las Pulgas, San Mateo, CA 94403). The SB, RF, and MS strains were kindly provided by Dr J. Yanke, Agriculture and Agri-Food Canada, Lethbridge, Alberta, Canada.

All bacterial cultures (1 mL of each bacterial inoculum + 9 mL of basal medium) were grown in a final volume of 10 mL (McDougall, 1948), which contained 30% clarified rumen fluid (filtrate by autoclaved rumen fluid) and 30% artificial saliva (vol/vol; pH 6.8), during a 48-h incubation at 39°C in Hungate tubes under either a CO_2_-H_2_ (20 mL; 50:50; vol/vol) atmosphere for SB, RF, MS, and their mixed culture, or a CH_4_-O_2_ (20 mL; 50:50; vol/vol) atmosphere (semi-anaerobic) for MC (Min et al., 2006). The artificial saliva was prepared with resazurin (Eastman Kodak), 0.2 mL; mineral solution numbers 1 and 2, 8 mL each; glucose, 0.2 g; cellobiose, 0.2 g; starch, 0.2 g; xylose, 0.2 g; trypticse peptone (BBL), 0.1 g; Na_2_CO_3_, 0.8 g; distilled water, 134 mL, in a final volume of 200 mL (McDougall, 1948). The pH was adjusted to pH 6.8 with 30% NaOH before dispensing to the medium. Cystein-HCl solution (2.5%) was then added (0.2 mL) to each tube after autoclaving.

A pure strain of MC was grown at 39°C in nitrate/mineral salts (NMS) medium, as described by Joergensen and Degn (1987). Preparation, distribution, and inoculation of a basal growth medium were carried out in an anaerobic hood (Coy Laboratory Products Inc., Grass Lake, MI). Standard gases, including CH_4_, H_2_, O_2_, and CO_2_, with a purity of 99.99% (GASCO Co., 320 Scariot Blvd., Oldsmar, FL 34677, USA), were used. The specific growth of the bacterial inoculum, based on optical density (OD) measured using a spectrophotometer (Milton Roy Co., Spectronic 20D, Rochester, NY) at 600 nm, was determined after 24 h of incubation at 39°C in Hungate tubes, as described by Min et al. (2006).

In Exp. 2 (ANKOM production system), linear dose levels of MC inoculum addition (0, 20, and 40 mL of inoculum replace with 50, 30, and 10 mL of artificial saliva, respectively) with 50 mL of mixed microbes freshly collected from cannulated steers in 250 mL incubation bottles with two basal diets (grain- and forage-based diets) on ruminal gas production, CH_4_ emissions, and fermentation rates were measured using an ANKOM gas production system. Before inoculating the bacterial strain, the inoculum was validated by measuring the bacterial growth and CH_4_ gas production, compared to the blank (only basal medium) without MC bacteria. Bottles containing only inoculum and buffer served as blanks. Each incubation was done in triplicate (*n* = 3). The anaerobic condition was preserved by continuously flushing the ANKOM bottle (ANKOM Technology Corp., Macedon, NY) headspace with CO_2_. For in vitro incubations, 6 g of mixed diets (% as-fed; Table 1) were placed in 250 mL ANKOM sample bottles containing 40 mL of (pH 5.80) and 50, 30, and 10 mL of artificial saliva (pH 6.8), respectively, to achieve a total volume of 90 mL in the incubation bottles.

**Table 1.** Ingredients and nutrient composition [% dry matter (DM)] of the experimental diets tested in vitro

Item^1^	Diet (% DM as-fed)			
	SFC	Alfalfa	Grain-based	Forage-based
**Ingredients**				
Steam-flaked corn	–	–	65.0	19.0
WDGS	–	–	10.0	10.0
Cottonseed meal	–	–	5.0	5.0
Alfalfa, ground	–	–	19.0	65.0
Urea	–	–	0.5	0.5
**Chemical composition**				
DM	90.4	91.0	91.5	91.7
CP	12.8	24.4	18.6	23.4
ADF	4.0	29.7	9.3	25.0
NDF	12.2	37.3	18.3	35.7
Lignin	1.2	7.0	3.1	5.5
NFC	70.7	22.9	52.2	26.8
Starch	61.9	0.8	41.1	10.1
CF	1.9	2.2	4.2	3.7
Ash	2.4	13.1	6.7	10.4
TDN	84.0	58.0	77.0	64.0

SFC=steam-flaked corn, WDGS = wet distiller’s grains plus solubles, DM=dry matter, CP = crude protein, ADF=acid detergent fiber, NDF=neutral detergent fiber, NFC=non-fiber carbohydrate (100-CP-NDF-CF-ash), CF =crude fat, TDN = total digestible nutrients [86.2 − (0.513 × % NDF) × 0.88].

In Exp. 3 (flux chamber system), two MC treatments were tested: control [0] and 40 mL of MC as an inoculum; initial OD = 0.82. These were inoculated onto two basal diets, and CH_4_ and fermentation end products were evaluated using continuous recirculating flux chamber systems. The study assessed the inclusion of MC in pure culture obtained from Exp. 1 or mixed populations of rumen microbes obtained from cannulated steers, using both grain- and forage-based diets, with a focus on bacterial growth rate, rumen fermentation rate, and ruminal CH_4_ production.

### Laboratory analyses

Following 48 h of fermentation, 1 mL samples from each incubator were transferred into 5 mL Falcon tubes and stored at −80°C for future VFA analysis. Fermentation products (alcohols, VFA, and aromatic-ring compounds) in frozen samples were analyzed by gas chromatography (Hewlett-Packard 6890, Agilent Technologies, Palo Alto, CA) as previously described (Varel et al., 2010). L-Lactate was analyzed using an autoanalyzer (Model 2700, Yellow Springs Instrument, Yellow Springs, OH). Additionally, two 10 mL samples were collected from each sample bag for analysis. Gas sample bags were sealed and separated from the module. Then the gas concentrations of CH_4_ and N_2_O were determined using an SRI gas chromatograph equipped with an electron capture detector (ECD) and a flame ionization detector (FID) (SRI Instruments, Torrance, CA; Bucha et al., 2025). The analyzers were calibrated with a known concentration of gas. Hydrogen sulfide (H_2_S) production was measured using a gas monitor (GX2012 model, RIKIN KEIKI Co. Ltd, Tokyo, Japan). Specific gas concentrations were plotted against total gas production to estimate overall gas emissions per hour or gas produced on a dry matter basis.

The nutrient content of ingredients for dry matter (DM), crude protein (CP), neutral detergent fiber (NDF), acid detergent fiber (ADF), crude fat (CF), and starch was analyzed by Dairy One Forage Testing Laboratory (Ithaca, NY). Analytical DM concentrations of diet samples were determined by oven drying at 105°C for 24 h (AOAC, 1998). Ash and minerals were analyzed according to the methods described by AOAC (2016). The in vitro dry matter disappearance rate (IVDMD) was calculated using the residual substances from the sample bottle, which were poured into pre-weighed 250 mL beakers. The beakers were then dried in a forced-air oven at 60°C for approximately 48 h and weighed. Concentrations of N were determined to estimate CP (N × 6.25) using an organic elemental analyzer (Flash 2000; CE Elantech Inc., Lakewood, NJ, USA; AOAC, 1998). Concentrations of NDF and ADF were sequentially determined using an ANKOM200/220 Fiber Analyzer (ANKOM Technology, Macedon, NY, USA) according to the manufacturer’s methodology, which was based on the method described by Van Soest and Robertson (1985). Sodium sulfite was used in the procedure for NDF determination and pre-treated with heat-stable amylase (Type XI-A from Bacillus subtilis; Sigma-Aldrich Corporation, St. Louis, MO, USA). Total digestible nutrient (TDN) concentration was calculated based on % NDF content [TDN = 86.2 – (% NDF × 0.513) × 0.88; Undersander et al., 1993]. Non-fiber carbohydrate (NFC) was calculated based on the % of CP, NDF, CF, and ash content (100-CP-NDF-CF-ash). The CF was measured by ether extract (AOAC, 1998) using a fat analyzer (XT20, ANKOM Technology).

### Data analysis and interpretation

All statistical analyses were performed using the GLM procedure (SAS Institute, 1987), examining factors such as diets, dose levels, and interactions between diet and dose levels. Data are presented as least-square means alongside the standard error of the mean (SEM). The least squares mean is reported throughout, and significance was considered at *P *< 0.05.

## Results and Discussion

### Nutrient composition of diets

The chemical composition of the dietary components and treatments is shown in Table 1. Among the main ingredients used in this study, steam-flaked corn had higher starch contents (61.9% DM) than alfalfa hay (0.8% as-fed). However, steam-flaked corn had lower NDF (12.2%) and ADF (4.0% DM) compared to alfalfa forage (37.3% and 29.7% DM, respectively). In grain-based diets, the contents of DM, CP, ADF, NDF, lignin, NFC, starch, and TDN were 90.4, 12.8, 4.0, 12.2, 1.2, 70.7, 61.9, and 84.0% as-fed, respectively, while forage-based diets contained 91.0, 24.4, 29.7, 37.3, 7.0, 22.9, 0.8, and 58% as-fed for DM, CP, ADF, NDF, lignin, NFC, starch, and TDN, respectively (Table 1). Therefore, increasing alfalfa inclusion in forage-based diets raised nutrient contents such as CP, ADF, NDF, and lignin. Meanwhile, within the grain-based diets, NFC, starch, and TDN increased as the amount of steam-flaked corn increased.

### Experiment 1: In vitro ruminal fermentation products and bacterial growth rate

**Figure 2.**
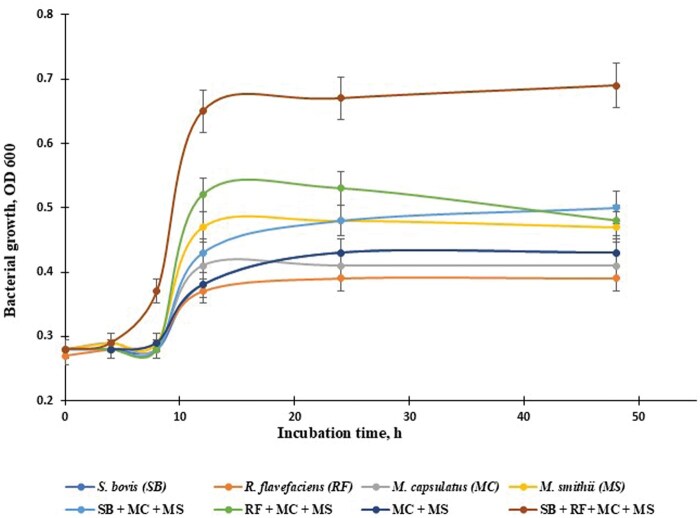
In vitro bacterial growth by predominant amylolytic (*Streptococcus bovis*; SB; -●-; blue filled circle) and cellulolytic (*Ruminococcus flavefaciens*; RF; -●-; light brown filled circle) rumen bacteria culture of mono or mixed-culture with *Methylococcus capsulatus* (MC; -●-; gray filled circle) and *Methanobrevibacter smithii* (MS; -●-; yellow filled circle). *M. smithii* was grown under an H_2_-CO_2_ (50:50, vol/vol) gas mixture, while *M. capsulatus* was grown under an O_2_-CH_4_ (20 mL; 50:50, vol/vol) gas mixture, and all other strains, including mixed-culture strains were grown in a 10 mL of final volume that contained clarified rumen fluid in a Hungate tube (n = 3). Mixed culture strains: SB+ MC + MS (-●-; light blue fileed circle), RF + MC + MS (-●-; light green filled circle), MC + MS (-●-), and SB + RF + MC + MS (-●-; brown filled circle).

Bacterial strains, cultured either alone or in combination with methanogens (*M. smithii*) and methanotrophic bacteria (*M. capsulatus*), along with cellulolytic and amylolytic bacterial combinations, are listed in Table 2. In monoculture, CH_4_ gas production was observed only from *M. smithii* with a similar maximum growth rate (Table 2). However, the highest growth rate was observed in mixed populations of ruminal microbes with all bacterial combinations (SB+RF+MC+MS; *P *< 0.05), intermediate for SB+MC+MS (P < 0.05), and lowest for MC+MS among co-cultures during a 48-h rumen incubation. The bacterial-specific growth was fastest for SB + FS + MC + MS (*P *< 0.01), intermediate for FS + MC + MS, *M. smithii*, and SB + MC + MS, and slowest for *M. capsulatus*, *S. bovis*, and *R. flavefaciens*, indicating that *M. capsulatus* tends to grow well when mixed populations of ruminal microbes, including methanogens (Figure 2). As a result, *S. bovis* was a major producer of lactic acid, but *M. smithii* mainly produced CH_4_ among the selected strains or in mixed populations of ruminal microbes.

**Table 2.** Bacterial maximum growth (48 h) and methane (CH_4_) gas production of amylolytic (*Streptococcus bovis;* SB) and cellulolytic (*Ruminococcus flavefaciens*; RF) rumen bacteria with culture of mono or mixed-cultures with *Methylococcus capsulatus* (MC) and *Methanobrevibacter smithii* (MS)

Item	**Growth rate** **OD_600_/48h**	Volatile fatty acids (VFA; mM)			A:P ratio	CH_4_ (µM/mL)
		Acetate	Propionate	Butyrate		
**Amylolytic bacteria**						
*Streptococcus bovis* (SB)	0.42^c^	14.0^b^	6.7^a^	1.8	2.1^b^	0.0
**Cellulolytic bacteria**						
*Ruminococcus flavefaciens* (RF)	0.39^c^	14.1^b^	6.6^a^	2.4	2.1^b^	0.0
**Methanogens**						
*Methanobrevibacter smithii* (MS)	0.41^c^	0.98	0.15^c^	0.03	6.5^a^	5.9
**Methanotrophs bacteria**						
*Methylococcus capsulatus* (MC)	0.47^c^	15.6^a^	6.4^a^	3.4	2.4^b^	0.0
**Cocultures**						
SB+MC+MS	0.50^b^	10.3^c^	4.7^b^	1.0	2.2^b^	0.0
RF+MC+MS	0.48^c^	13.8^b^	4.7^b^	1.1	2.9^b^	0.0
MC + MS	0.43^c^	14.2^b^	4.8^b^	1.0	3.0^b^	0.0
SB+RF+MC+MS	0.69^a^	11.9^c^	5.6^b^	1.2	2.1^b^	0.0
**SEM**	0.026	0.43	0.12	0.25	1.78	0.01

a–c Within a column, means without a common superscript letter differ (*P *< 0.05). *M. smithii* was grown under H_2_-CO_2_ (50:50, vol/vol) gas mixture. In contrast, *M. capsulatus* was grown under O_2_-CH_4_ (60:40, vol/vol) gas mixture, and all the strains, including co-culture strains, were grown in 10 mL of basal medium that contained clarified rumen fluid (5%) in a Hungate tube.

In the absence of an external supply of H_2_ and CH_4_, *M. smithii* and *M. capsulatus* could not grow as monocultures in the growth medium (data not shown). Therefore, *M. capsulatus* was cultured under elevated dissolved O_2_-water (23 ppm), along with 20 mL of exogenous pure O_2_ and CH_4_ gases (50:50; vol/vol). Methanogens were grown in slightly oxygenated water (3.0 ppm) with 20 mL of a 50:50 (vol/vol) mixture of H_2_ and CO_2_ supplied. When a minor increase in dissolved O_2_-water (from 4.32 ppm, the O_2_ content of tap water, to 5 ppm; semi-anaerobic) was introduced as a fermentation substrate in the growth medium—affecting all mono- and co-cultures—longer lag times ranging from 5 to 8 h were observed before growth commenced, and maximum growth reached less than 0.5 OD, compared to all other combinations. This suggests that even a slight elevation in dissolved O_2_ in water may inhibit bacterial growth.

### Exp. 2

#### ANKOM gas production system

**Figure 3.**
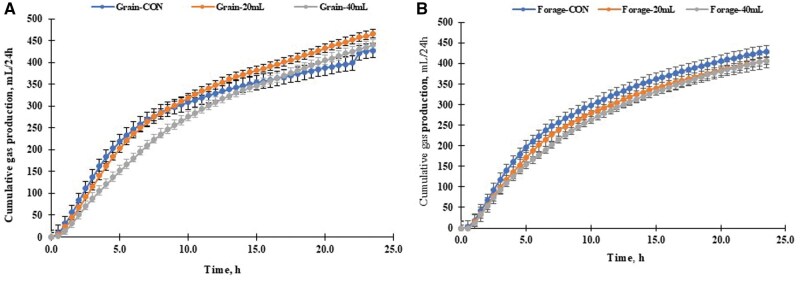
Effects of direct-fed methylotroph *Methylococcus capsulatus* (Bath) bacterial (±) inoculum (initial OD = 0.82) mixed-cultures with mixed populations of rumen microbes (pH 6.8) in the grain- (a) or forage-based (b) diets (*n* = 3) on cumulative gas production during the 24 h in vitro fermentation. (a) Grain-CON (control; -●-; blue filled circle), grain-20 mL (-●-; orange filled cirlce), and grain-40 mL (-●-; gray filled circle). (b) Forage-CON (control; -●-; blue filled cirlce), forage-20 mL (-●-; orage filled cirlce), and forage-40 mL (-●-; gray filled cirlce).

Results from ruminal pH, IVDMD, and gas production parameters are shown in Table 3 and Figure 3. There were no differences between *M. capsulatus* addition and basal diets in total gas production (Table 3) and cumulative gas production (Figure 3). However, ruminal pH and IVDMD (%) in the forage-based diet, along with potential gas (a + b) production in the grain-based diet, increased with higher levels of *M. capsulatus*. In contrast, the rate of gas production (c) decreased as *M. capsulatus* levels increased in both grain- and forage-based diets. A significant interaction was observed between *M. capsulatus* and basal diets for pH (*P *< 0.001), IVDMD (*P *< 0.03), and potential gas production (*P *= 0.08). Overall, the average pH (*P *< 0.001) was higher in forage-based diets compared to grain-based diets, while the rate of gas (c; *P *< 0.03) was lower in forage-based diets than in grain-based diets. Increasing *M. capsulatus* levels resulted in higher average ruminal pH, IVDMD, and potential gas production; however, the rate of gas production decreased as *M. capsulatus* levels increased.

**Table 3.** Ankum gas production system-mixed populations of ruminal microbes

Item	*Methylococcus capsulatus* (mL)							***P*-value**		
	Grain-based			Forage-based			SEM	Diet	Dose	INT
	0	20	40	0	20	40				
**Rumen pH**	5.1	5.1	5.0	5.1^b^	5.5^a^	5.5^a^	0.026	0.01	0.001	0.001
**IVDMD, %**	44.7	44.1	46.8	37.6^c^	40.6^b^	44.2^a^	0.78	0.01	0.001	0.03
**Ruminal gas production**										
Total gas production, mL/24h	427.1	465.0	439.4	418.7	393.9	406.5	26.28	0.16	0.97	0.62
Total gas production, mL/g DM	69.7	75.9	71.7	68.3	64.3	66.3	4.35	0.16	9.97	0.62
Gas production parameters										
Potential gas production (*a* + *b*)	306.3^b^	442.7^ab^	594.5^a^	425.1	478.9	504.8	41.18	0.54	0.01	0.08
Rate of gas production (*c*)	2.09^a^	1.54_ab_	0.59^b^	0.63^a^	0.08^ab^	0.06^b^	0.47	0.03	0.05	0.25
**Average**	**Grain-based**	**Forage-based**	**SEM**	***P*-value**		**Dose level**			**SEM**	***P*-value**
**pH**	5.06^b^	5.40^a^	0.01	0.001		5.1^b^	5.3^a^	5.3^a^	0.02	0.01
**IVDMD**	45.2^a^	40.8^b^	0.45	0.001		41.1^b^	42.4^ab^	45.5^a^	0.56	0.001
**Total gas, mL/24h**	443.8	408.6	16.84	0.15		422.9	429.4	422.9	21.9	0.83
**Total gas, g DM**	72.4	66.3	2.71	0.16		68.9	70.1	68.9	3.44	0.83
**Gas production parameters**										
Potential gas production (*a* + *b*)	447.8	469.6	25.08	0.54		365.7^c^	460.8^b^	549.7^a^	29.11	0.01
Rate of gas production (*c*)	1.230^a^	0.260^b^	0.296	0.03		1.35^a^	0.814^ab^	0.065^b^	0.337	0.02

In vitro total gas production and kinetics of gas production as a function of the addition of various levels of *Methylococcus capsulatus* (BATH) with two different basal diets (Exp. 2).

1 The values of *a*, *b*, and *c* were constants of the exponential equation (Eq. 1; Orskov and McDonald, 1979), where *a* = gas production at time 0, *b* = the proportion of gas production during time *t*, and *c* = the rate of gas production of the *b* fraction. IVDMD = in vitro dry matter disappearance rate, SEM = Standard error of mean. The 250 mL ANKOM sample bottles were used which contained 50 mL of pooled ruminal fluid (pH 5.6) and 50 mL of artificial saliva (pH 6.8; Min et al., 2005).

a–c Means within row treatment with a different superscript differ at *P *< 0.05.

#### In vitro ruminal gas production

In vitro, ruminal greenhouse gas (GHG) emissions as a function of adding *M. capsulatus* and basal diets are shown in Table 4. In the presence of *M. capsulatus*, there was a decrease (*P *< 0.01) in CH_4_ production (either as a percentage or grams per DM) with up to 40 mL of *M. capsulatus* added. However, substrates with basal diets (grain-based and forage-based) combined with *M. capsulatus* exhibited greater (*P *< 0.001) N_2_O emissions per gram of DM (µg/g DM) or total N_2_O produced over 24 h than the control. A significant interaction was observed between *M. capsulatus* and basal diets for N_2_O production (*P *< 0.001). Across the diets, average CH_4_ and CO_2_ (% or mL 24 h) and N_2_O (ppm; *P *< 0.01) were greater for the forage-based diet than for the grain-based diets. Average ruminal CH_4_ production was decreased (*P *< 0.01) with increasing *M. capsulatus*, but N_2_O production was increased (*P *< 0.001) with increasing *M. capsulatus*.

**Table 4.** Ankum gas production system-mixed populations of ruminal microbes

Item	*Methylococcus capsulatus*							***P*-value**		
	Grain-based			Forage-based			SEM	Diet	Dose	INT
Ruminal gas production	0	20	40	0	20	40				
**Methane (CH_4_)**										
CH_4_, %	15.1^a^	11.3^ab^	8.7^b^	20.5^a^	17.6^ab^	11.4^b^	1.89	0.02	0.01	0.64
CH_4_, mL/24h	64.6^a^	51.3^ab^	39.0^b^	86.5^a^	69.5^ab^	66.3^b^	4.35	0.16	0.02	0.62
CH_4,_ mL/g DM	10.5^a^	8.3^ab^	6.4^b^	14.0^a^	11.3^ab^	7.6^b^	1.47	0.09	0.02	0.75
CH_4_, mg/g DM	7.5^a^	5.9^ab^	4.5^b^	10.0^a^	8.0^ab^	5.4^b^	1.05	0.09	0.02	0.75
**Carbon dioxide (CO_2_)**										
CO_2_, %	30.7	25.7	30.2	48.4	52.5	48.3	5.16	0.01	0.99	0.71
CO_2_, mL/24h	131.1	119.9	132.9	203.7	206.6	196.4	23.80	0.01	0.98	0.91
**Nitrous oxide (N_2_O)**										
N_2_O, ppm	5.86^b^	20.8^ab^	163.7^a^	3.7^b^	67.2^ab^	458.6^a^	38.48	0.01	0.001	0.01
N_2_O, mL/24h	0.003	0.01	0.07	0.02	0.03	0.19	0.014	0.01	0.001	0.01
N_2_O, µL/g DM	0.43^b^	1.6^ab^	11.7^a^	0.3^b^	4.3^ab^	30.3^a^	2.44	0.01	0.001	0.01
N_2_O, µg/g DM	0.8^b^	3.1^ab^	23.0^a^	0.5^b^	8.5^ab^	59.5^a^	4.80	0.01	0.001	0.01
Hydrogen sulfate (H_2_S)										
H_2_S, ppm	3.1	0.5	0.7	3.6	1.1	13.9	6.68	0.36	0.57	0.48
H_2_S, mL/24h	0.001	0.002	0.003	0.007	0.004	0.005	0.002	0.38	0.50	0.47
**Average**	**Diet**					**Dose level**				
	**Grain-based**	**Forage-Based**	**SEM**	***P*-value**		**0**	**20**	**40**	**SEM**	***P*-value**
**CH_4_, %**	11.8^b^	16.5^a^	1.17	0.02		17.8^a^	14.5^ab^	10.1^b^	1.48	0.01
**CH_4_, mL/24h**	51.7	67.3	5.65	0.09		75.3^a^	60.4^ab^	42.8^b^	7.16	0.01
**CH_4_, mL/g DM**	8.4	10.9	0.12	0.09		12.3^a^	9.8^ab^	6.9^b^	1.16	0.01
**CH_4_, mg/g DM**	6.1	7.8	0.68	0.09		8.8^a^	7.0^ab^	4.9^b^	0.83	0.001
**CO_2_, %**	28.9^b^	49.8^a^	3.21	0.001		39.6	39.1	39.3	4.08	0.96
**CO_2_, mL/24h**	128.0^b^	202.2^a^	14.84	0.01		167.4	163.3	164.6	18.8	0.88
**N_2_O, ppm**	63.4^b^	176.5^a^	23.99	0.01		4.8^b^	43.9^ab^	311.1^a^	30.42	0.001
**N_2_O, mL/24h**	0.03^b^	0.07^a^	0.009	0.01		0.002^c^	0.02^b^	0.13^a^	0.011	0.001
**N_2_O, µL/g DM**	4.6^a^	11.6^b^	1.52	0.01		0.34^c^	2.3^b^	20.9^a^	1.93	0.001
**N_2_O, µg/g DM**	9.0^b^	22.8^a^	2.99	0.01		0.7^c^	5.8^b^	41.3^a^	3.80	0.001
**H_2_S, ppm**	1.42	6.2	3.4	0.36		3.4	0.8	7.2	4.31	0.56
**H_2_S, mL/24h**	0.001	0.002	0.62	0.38		0.01	0.003	0.03	0.001	0.56

In vitro methane gas production as a function of adding various levels of *Methylococcus capsulatus* (BATH) with two different basal diets (Exp. 2).

SEM = Standard error of mean. The 250 mL ANKOM sample bottles were used which containing 50 mL of pooled ruminal fluid (pH 5.6) and 50 mL artificial saliva (pH 6.8; Min et al., 2005).

a–c Means within row treatment with a different superscript differ at *P *< 0.05.

#### Rumen fermentation profiles

Rumen fermentation profiles are shown in Table 5. Total VFA and iso-butyrate levels were similar across treatments, but acetate concentration decreased (*P *< 0.01) with increasing *M. capsulatus*. Additionally, propionate, butyrate, and valerate were lower (*P *< 0.01) for the 20% *M. capsulatus* group compared to the control and the 40 mL *M. capsulatus* group. Consequently, the A: P ratio was higher for the 20 mL *M. capsulatus* group than for the control or 40 mL *M. capsulatus* group. A significant interaction was observed between *M. capsulatus* and basal diets for valerate and A: P ratio (*P *< 0.001).

**Table 5.** The effect of methylotroph *Methylococcus capsulatus* bacterial inoculum co-cultures with mixed populations of rumen microbes (pH 6.8) in grain- or forage-based diets (*n* = 3) on in vitro ruminal volatile fatty acids (VFA) production

Item	*Methylococcus capsulatus*							***P*-value**		
	Grain-based			Forage-based			SEM	Diet	Dose	INT
	0	20	40	0	20	40				
**VFA, mol 100 mol^-1^**										
**Total VFA, mM**	107.9	96.6	103.9	112.7	106.4	109.8	3.26	0.02	0.05	0.73
**Acetate**	59.6^a^	55.4^ab^	54.6^b^	70.1^a^	66.8^ab^	65.9^b^	1.84	0.01	0.05	0.96
**Propionate**	21.5^a^	18.4^b^	21.3^a^	20.3^a^	18.8^b^	20.1^a^	1.25	0.23	0.01	0.32
**Iso-butyrate**	0.8	0.7	0.8	1.1	1.0	1.1	0.035	0.01	0.13	0.92
**Butyrate**	19.0^a^	16.2^b^	20.1^a^	14.1^ab^	13.7^b^	14.6^a^	0.71	0.01	0.001	0.25
**Iso-valerate**	1.5	1.3	1.6	1.9^ab^	1.8^b^	2.1^a^	0.08	0.01	0.01	0.71
**Valerate**	4.0^b^	3.3^c^	4.6^a^	4.1^b^	3.8^c^	4.4^a^	0.09	0.06	0.01	0.02
**A:P ratio**	2.8^b^	3.0^a^	2.6^c^	3.4^ab^	3.5^a^	3.3^b^	0.035	0.01	0.001	0.05
						^**Dose level**^				
**Average**	**Grain-based**	**Forage-based**	**SEM**	***P*-value**		**0**	**1**	**2**	**SEM**	***P*-value**
**Total VFA, mM**	102.8^b^	109.6^a^	3.15	0.05		110.3^a^	101.2^b^	106.9^ab^	3.09	0.01
**Acetate**	56.5^b^	67.6^a^	1.06	0.001		64.8^a^	61.1^ab^	60.3^b^	1.30	0.02
**Propionate**	20.4	19.8	0.34	0.24		20.9^a^	18.6^b^	20.7^a^	0.42	0.01
**Iso-butyrate**	0.8^b^	1.1^a^	0.02	0.001		0.9	0.9	0.9	0.05	0.25
**Butyrate**	18.4^a^	13.3^b^	0.45	0.001		16.3^a^	14.4^b^	16.9^a^	0.49	0.01
**Iso-valerate**	1.5^b^	2.0^a^	0.04	0.01		1.7^b^	1.5^c^	1.9^a^	0.05	0.01
**Valerate**	3.9	4.0	0.05	0.07		4.6^a^	3.6^b^	4.5^a^	0.06	0.01
**A:P ratio**	2.8^b^	3.4^a^	0.02	0.001		3.1^ab^	3.3^a^	2.9^b^	0.03	0.001

Values within rows followed by superscript letters (^a, b, c^) indicate significant difference (*P *< 0.05) for grain-based and forage-based diets. Treat = treatment effect.

VFA = volatile fatty acids; A: P = acetate: propionate ratio.

Across the diets, the average total VFA (Table 6), acetate, iso-butyrate, iso-valerate, and A:P ratio (*P *< 0.05) were higher for the forage-based diet than for grain-based diets. The average total VFA and acetate production decreased (*P *< 0.01) with increasing *M. capsulatus*, but propionate, butyrate, isovalerate, and valerate concentrations were lower (*P* < 0.01) for 20 mL of *M. capsulatus* compared to the control or 40 mL of *M. capsulatus*.

**Table 6.** The effect of methylotroph *Methylococcus capsulatus* (Bath) bacterial (±) inoculum (initial OD = 0.82) co-cultures with mixed populations of rumen microbes (pH 6.8) in the grain- (a) or forage-based (b) diets (*n* = 3) on in vitro ruminal pH and gas production with either grain-based or forage-based diets

Rumen pH and fermentation product	Grain-based diet		Forage-based diet		Average		SEM	Diet	Treat	Diet × Treat
	Bath (initial OD = 0.82)		Bath (initial OD = 0.82)		Grain-based diet	Forage-based diet				
	**Control ** **0 mL**	40 mL	**Control ** **0 ppm**	40 mL						
pH	5.1	5.1	5.4	5.5	5.1^b^	5.5^a^	0.04	0.01	0.49	0.52
**VFA, mol 100 mol^−1^**										
Total VFA	90.6^a^	84.6^b^	93.1	93.8	87.6^b^	93.4^a^	0.63	0.01	0.01	0.01
Propanol	0.0	0.01	0.002	0.01	0.002	0.005	0.003	0.30	0.11	0.67
Butanol	0.09^ab^	0.18^a^	0.02^b^	0.12^ab^	0.14	0.10	0.044	0.16	0.08	0.88
Acetate	59.6^c^	54.6^c^	70.1^a^	66.0^ab^	57.1^b^	68.0^a^	1.87	0.001	0.04	0.81
Propionate	21.5	21.3	20.4	20.2	21.4	20.3	0.60	0.09	0.76	0.98
Iso-butyrate	0.8^b^	0.8^b^	1.1^a^	1.1^a^	0.80^b^	1.11^a^	0.034	0.001	0.24	0.69
Butyrate	19.0^a^	20.0^a^	13.6^b^	13.8^b^	19.5^a^	13.7^b^	0.70	0.001	0.42	0.57
Iso-valerate	1.5^b^	1.6^b^	1.9^ab^	2.1^a^	1.6^b^	2.0^a^	0.08	0.001	0.20	0.46
Valerate	4.0^b^	4.6^a^	4.1^b^	4.5^a^	4.3	4.3	0.07	0.88	0.01	0.28
Heptanoate	0.05^b^	0.07^b^	0.07^b^	0.13^a^	0.06^b^	0.10^a^	0.09	0.01	0.01	0.02
Phenol	0.01	0.01	0.01	0.02	0.01	0.02	0.003	0.19	0.56	0.87
p-cresol	0.08^b^	0.08^b^	0.16^a^	0.14^a^	0.08^b^	0.15^a^	0.008	0.01	0.26	0.25
Indole	0.03	0.03	0.02	0.03	0.03	0.02	0.002	0.09	0.26	0.10
Skatole	0.0	0.001	0.001	0.001	0.001	0.001	0.0001	0.81	0.52	0.45
Benzoate	0.06	0.04	0.05	0.05	0.05	0.05	0.009	0.94	0.35	0.26
Phenylacetate	0.19^b^	0.13^b^	0.40^a^	0.40^a^	0.16^b^	0.40^a^	0.034	0.01	0.35	0.44
Phenylpropionate	0.50^b^	0.54^b^	0.71^a^	0.75^a^	0.52^b^	0.73^a^	0.01	0.001	0.01	0.93
Lactate	2.7^a^	0.03^b^	0.05^b^	0.05^b^	1.4^a^	0.05^b^	0.091	0.19	0.18	0.19
Ethanol	4.2^b^	6.1^a^	2.7^c^	2.1^c^	5.1^a^	2.4^b^	0.537	0.01	0.26	0.04
A:P ratio	2.77^d^	2.56^c^	3.44^a^	3.27^b^	2.67^b^	3.6^a^	0.032	0.001	0.001	0.57

Values within rows followed by superscript letters (^a, b, c^) indicate significant difference (*P *< 0.05) for grain-based and forage-based diets. Treat =bath treatment effect.

VFA = volatile fatty acids; A:P = acetate:propionate ratio.

### Exp. 3. Continuous gas production system

**Figure 4.**
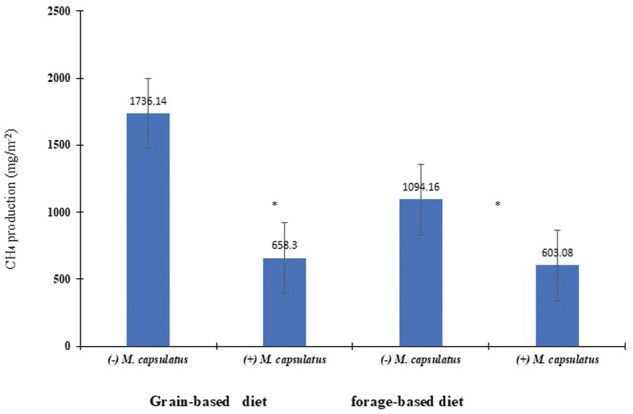
Effects of direct-fed methylotroph *Methylococcus capsulatus* (Bath) bacterial (±) inoculum (initial OD = 0.82) co-cultures with mixed populations of ruminal microbes (pH 6.8) in grain-based (a) or forage-based (b) diets (*n* = 3) on methane gas (CH_4_) emission (mg/m^−2^) during the 24 h in vitro fermentation in a semi-anaerobic batch culture system. * Means with different letters differ significantly (*P *< 0.05).

In vitro CH_4_ production as a function of adding *M. capsulatus* and basal diets is shown in Figure 4. Without *M. capsulatus*, there was a significant increase (*P *< 0.05) in total CH_4_ production for grain-based (1735.14 mg/m^−2^) and forage-based diets (1094.16 mg/m^−2^). However, with *M. capsulatus*, these trends significantly decreased (*P *< 0.001) in both grain-based and forage-based diets.

## Discussion

The role of CH_4_ as a GHG and the contribution of bacteria to the production (methanogenesis) and sink (CH_4_ oxidation) of CH_4_ are examined. Because bacterial activity influences global CH_4_ production and reduction, it is essential to understand both the organisms involved and their responses to short- and long-term environmental changes. The primary objectives of this study were to investigate the impact of *M. capsulatus* inoculation on the growth rates of other bacteria, fermentation end products, CH_4_ gas production, and changes in the rumen microbial community associated with methanogenesis. *M. capsulatus*, a methanotrophic bacterium, is prokaryotes that metabolize CH_4_ as its only source of carbon and energy to survive. In this study, CH_4_-producing archaea, *M. smithii*, were chosen as a suitable CH_4_ source for *M. capsulatus*, along with coculture with other rumen bacterial strains, to explore the potential roles of microbial activities associated with methanogenesis and methanotroph pathways.

When an elevated O_2_-water supply (from 0.5 ppm [tap water O_2_ content] to 5 ppm O_2_) was added to the growth medium for *M. capsulatus* and all other strains, all bacterial strains exhibited longer lag times, ranging from 5 to 8 h, before initiating growth. They also reached a lower maximum growth rate (< 0.5 OD value) compared to all other conditions, indicating that elevated O_2_-water supply may inhibit bacterial growth, including methanogens (acting as an inhibitor). Patel and Hoare (1971) reported that the doubling time of *M. capsulatus* was 6 to 8 h when using CH_4_ and methanol as sole carbon sources. In the present study, with a one-time supply of CH_4_ (20 mL of pure CH_4_ and O_2_), the growth rate of *M. capsulatus* was slower than the published data (Joergensen and Degn, 1987; Patel and Hoare, 1971), which used continuous culture with dissolved CH_4_ supply (23 mL/min gas flow containing 1.023 M CH_4_ and 0.978 M O_2_), because CH_4_ became the growth-limiting factor. However, the apparent saturation constant, KS, for CH_4_ utilization in resting methanotrophic bacteria has been found by several authors to vary from 0.8 µM to 66 µM (Lamb and Carver, 1980; Joergensen and Degn, 1983; Megraw and Knowles, 1987), and further study is required.

The rumen hosts one of the most efficient microbial ecosystems for breaking down plant cell walls, ultimately producing CH_4_ gas. However, the role of dominant amylolytic and cellulolytic bacteria, in association with methanogens and methanotrophs, in symbiotic CH_4_ production remains unclear. In this study, when mixed populations of ruminal microbes with *M. capsulatus* and *M. smithii* were studied, bacterial growth rates were lower than in other mixed populations of ruminal microbe combinations (MC + MS + RF or MC + MS + SB), suggesting that *M. smithii* needs an external H_2_ supply for growth. Increases in methotroph populations were only detectable at CH_4_ concentrations above 11,000 ppm (Bender and Conrad, 1995). The highest rates of CH_4_ oxidation occurred when the vertical profiles of CH_4_ and O_2_ overlapped (Hanson and Hanson, 1996). Min et al. (2006) reported that most cellulolytic bacterial strains, such as *R. flavefaciens* and *Fibrobacter succinogenes*, produce H_2_ (1.68 and 6.4 µM H_2_/mL, respectively) in the rumen and support CH_4_ production for *M. smithii* growth when mixed-cultured with those cellulolytic strains, utilizing H_2_ to reduce CO_2_ to CH_4_, as described by Latham and Wolin (1977). This finding is consistent with bacterial growth rates observed in this and other studies (Miller and Wolin, 1973; Wolin et al., 1997).

Methanogenic archaea form a unique group of strictly anaerobic microorganisms that derive their energy for growth from the reduction of CO_2_ and H_2_ or other simple compounds (e.g., acetate, formate, methanol, and alkylamines) to CH_4_ (Jarrel, 1985). It has been reported that the threshold concentration of H_2_ is around 30–100 ppm for methanogens (Cord-Ruwisch et al., 1988). Additionally, it has been noted that *M. smithii* is significantly enriched with metabolic genes involved in CO_2_ and H_2_ utilization during methanogenesis (Samuel et al., 2007). Therefore, it can be hypothesized that rumen methanogen culture, combined with cellulolytic bacterial strains that produce H_2_ as an electron donor, could promote more synergistic effects and foster a symbiotic relationship for methanogenesis.

The main sources of ruminal gases are microbial fermentation and acidification of bicarbonate. The major components of ruminal gases are CO_2_, CH_4_, O_2_, N_2_, H_2_, and H_2_S (Clarke and Reid, 1974). Results from the present experiment show that *M. smithii* produced the most CH_4_ among strains. At the same time, no CH_4_ was detected when mixed-cultured with *M. capsulatus*, indicating that *M. capsulatus* could utilize most of the CH_4_ when cocultured with *M. smithii*. Therefore, a comparison of effects between methane-utilizing bacteria and their mixed culture with methanogens might provide the biological answer to the CH_4_ sink related to the methanogenesis pathways. However, our understanding of the physical and chemical responses to interspecies interactions and the *in vivo* CH_4_ sink is unclear.

### Methanogenesis, methanotrophs, and microbial ecosystem

**Figure 5.**
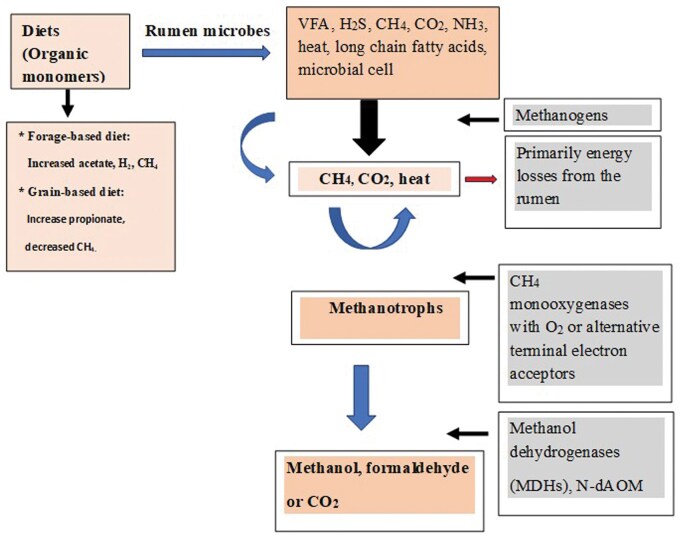
Schematic microbial fermentation of diets, methanogenesis, and methanotrophs’ biological activities in the rumen and environment. CH_4_ = methane; H_2_ = hydrogen; NH_3_ = ammonia; O_2_ = oxygen; VFA = volatile fatty acids; H_2_S = hydrogen sulfide; N-dAOM = nitrite-dependent anaerobic oxidation of methane. Boxes with bold solid lines are potential targets for suppressing CH_4_ emissions. The host animal uses the VFA, NH_3_, microbial cells, and long-chain fatty acids. H_2_S, CH_4_, and heat are the primary energy losses from the rumen. *Sources*: Conrad et al. (1985), Guerrero-Cruz et al. (2021), Keltjens et al. (2014), Latham et al. (1977), Miller and Wolin (1973), Min et al. (2006), Ungerfeld (2015), and Tokura et al. (2006).

To enhance understanding, a summary of methanogenesis, methanotrophs, and microbial fermentation of dietary components in the rumen and environment is presented in Figure 5. It has been noted that feeding concentrate diets high in starch generally lowers CH_4_ emissions (g/d and g/kg DMI); whereas high forage-based diets in beef and dairy cattle result in increased CH_4_ emissions associated with H_2_ production from cellulose-digesting bacteria and protozoa (Ungerfeld, 2015; Min et al., 2022), which is similar to the current study. Ruminal methanogens utilize reducing equivalents produced by fermentative microflora (primarily H_2_-producing microorganisms), including *Ruminococcus albus*, *R. flavefaciens*, *Neocalimastrix* spp., *Desulfovibrio*, and ciliate protozoa (Conrad et al., 1985; Tokura et al., 2006; Ungerfeld, 2015). According to Min et al. (2006), *R. albus* and *R. flavefaciens* (cellulolytic bacteria) ­produce the most H_2_ among purified strains and sustain CH_4_ production when mixed cultured with *M. smithii*, which utilizes H_2_ to reduce CO_2_ to CH_4_ (Latham and Wolin, 1977), a finding also supported by Miller and Wolin (1973) and the current study. Syntrophic cooperation between H_2_ consumers (e.g., methanogens) and H_2_ producers influences the overall fermentation balance of the primary substrate, resulting in improved energy utilization (Conrad et al., 1985).

Methanotrophs (called methanophiles) are prokaryotes that oxidize/metabolize CH_4_ as their source of carbon and energy (Oremland and Culbertson, 1992). The CH_4_ is oxidized by methanotrophic microorganisms using oxygen (O_2_; aerobic) or alternative terminal electron acceptors (anaerobic) (Guerrero-Cruz et al., 2021). The oxidation of CH_4_ is catalyzed by methane monooxygenases (pMMO and sMMO) belonging to the phyla Proteobacteria and Verrucomicrobia, while anaerobic CH_4_ oxidation is mediated by anaerobic methanotrophs within the bacteria and archaea domains (van Teeseling et al., 2014; Dedysh and Knief, 2018; Figure 5). An aerobic methanotrophic bacterial enzyme oxidizes CH_4_ to methanol, which is further oxidized by methanol dehydrogenases (MDHs) to formaldehyde (Keltjens et al., 2014). However, the anaerobic oxidation of CH_4_ (nitrogen-dependent anaerobic methane oxidation; N-dAOM) is coupled to the reduction of organic electron acceptors, such as manganese, nitrate, nitrite, iron, and sulfate (Guerrero-Cruz et al., 2021; Haroon et al., 2013).

We have realized that methanotrophs may play a significant role in the global CH_4_ budget, and therefore, in mitigating the impact of CH_4_ on global warming. In the present study, they oxidized most of the CH_4_ produced in facultative fermentation conditions before it reached the headspace of the in vitro fermenter. However, predicting symbiotic relationships between methanotrophs and methanogen activity remains challenging due to the differences in biochemical processes (aerobic vs. anaerobic). Therefore, anaerobic CH_4_-oxidizing organisms are essential, yet we know little about them.

## Conclusion

Results from current studies have shown that *Methanobrevibacter smithii* produces the most CH_4_ among the strains. At the same time, no CH_4_ was detected when mixed cultured with *M. capsulatus*, indicating that *M. capsulatus* could utilize most of the CH_4_ produced when cocultured with *Methanobrevibacter smithii* and other bacterial strains. This data supported subsequent experiments, showing that in the presence of *M. capsulatus*, CH_4_ production decreased as the amount of *M. capsulatus* increased. It can be concluded that adding *M. capsulatus* to basal diets significantly reduces CH_4_ emissions compared to the control. Therefore, comparing the effects of methane-utilizing bacteria inoculation might provide a biological solution to the CH_4_ sink related to methanogenesis pathways in ruminants. The use of methanogenesis or methanotrophs in climate change mitigation is essential for advancing current chemical-based industrial practices and developing bio-based solutions. Future research on innovative engineering approaches that utilize methane waste from human-made ecosystems could usher in a new era of methanotrophic waste management and production systems that achieve near-zero net emissions, helping to balance our impact on the carbon cycle.
